# Supplementary material for: A Refined Single Cell Landscape of Haematopoiesis in the Mouse Foetal Liver
Source: J Dev Biol. 2023 Mar 23;11(2):15. doi: 10.3390/jdb11020015 (PMC10123705; doi:10.3390/jdb11020015)
Supplement: Supplementary file 1 [file jdb-11-00015-s001.zip › jdb-2208475-supplementary.pdf]

Supplemental Materials

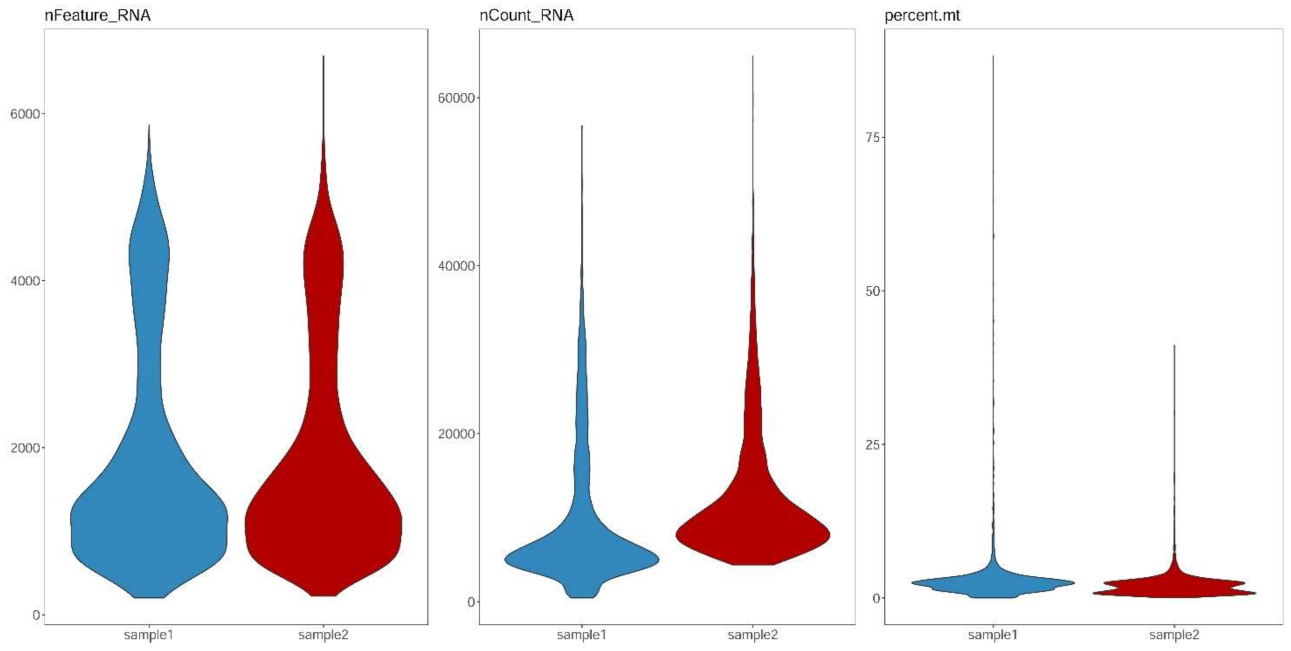

Figure S1. Quality check of the E12.5 mouse liver scRNA-seq dataset. Violin plots visualise the distribution of the total number of expressed genes per cell (left panel), the total number of transcripts detected per cell (central panel) and the percentage of mitochondrial gene expression (right panel) in the two replicates.

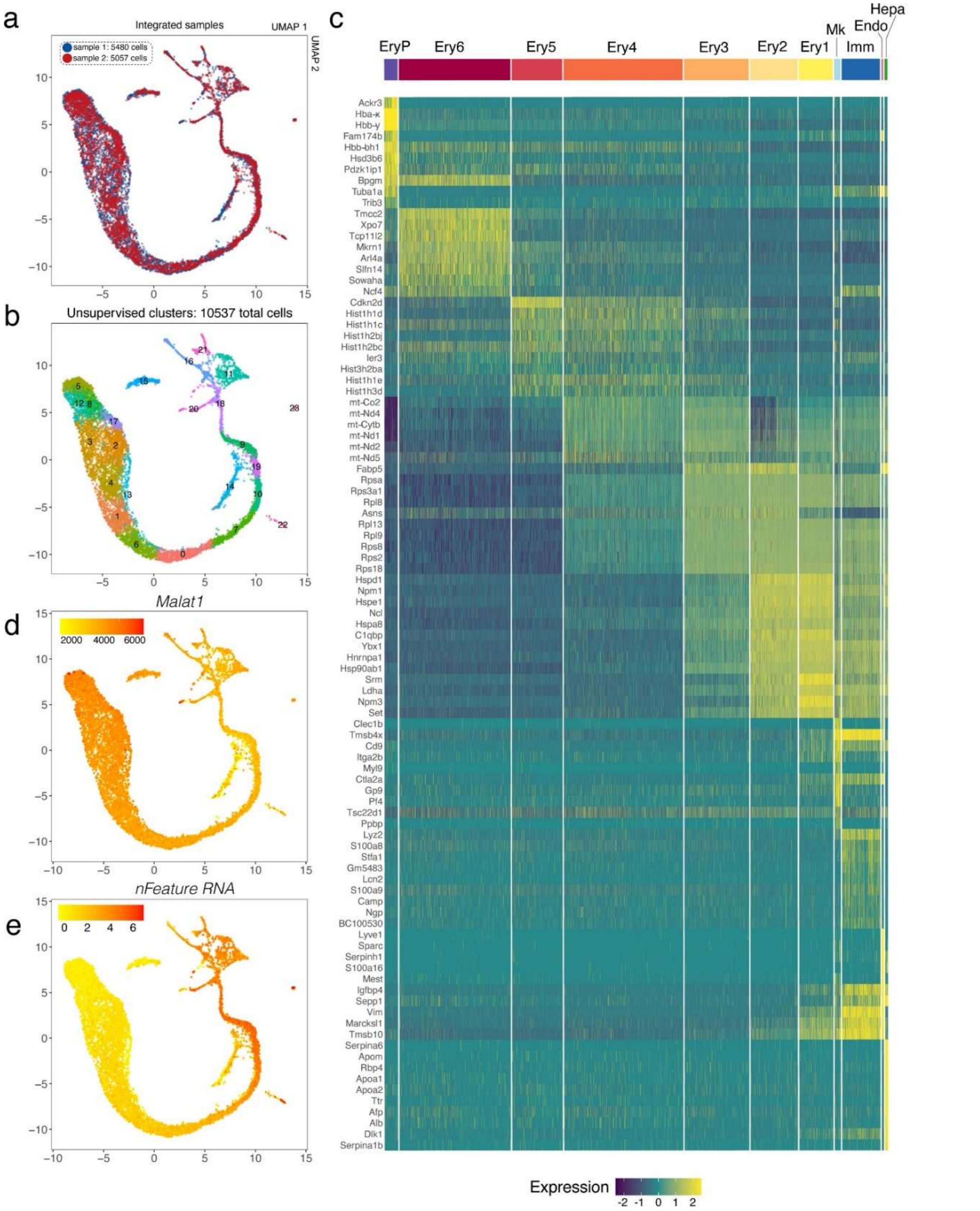

Figure S2. Characterisation of the E12.5 mouse liver scRNA-seq dataset. (**a**) A UMAP plot visualises the distributions of the two technical replicates from the same liver sample. (**b**) Unsupervised clustering of cell types into clusters 1-23. (**c**) A heatmap plot of differentially expressed genes (DEGs) among the curated cell clusters. (**d**) A UMAP plot visualises *Malat1*expression; *Malat1*-negative cells in the Ery2 cluster likely represent apoptotic bodies. (**e**) A UMAP plot visualises the number of expressed genes per cell.

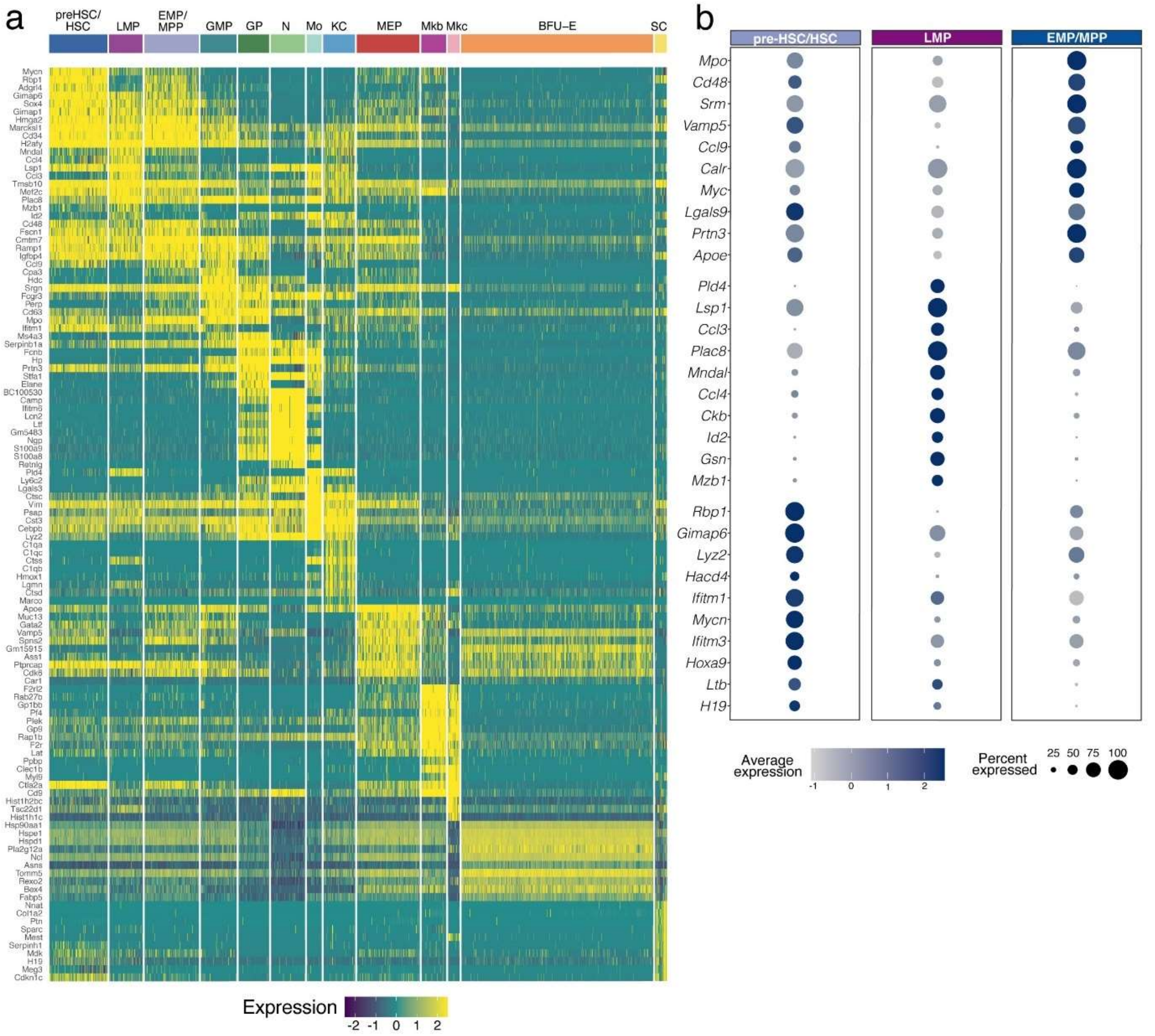

Figure S3. Heatmap of differential gene expression in haematopoietic cell populations of the E12.5 mouse liver. (**a**) A heatmap plot shows DEGs among the haematopoietic cell clusters.
(**b**) A bubble plot shows DEGs among the haematopoietic progenitor subclusters.

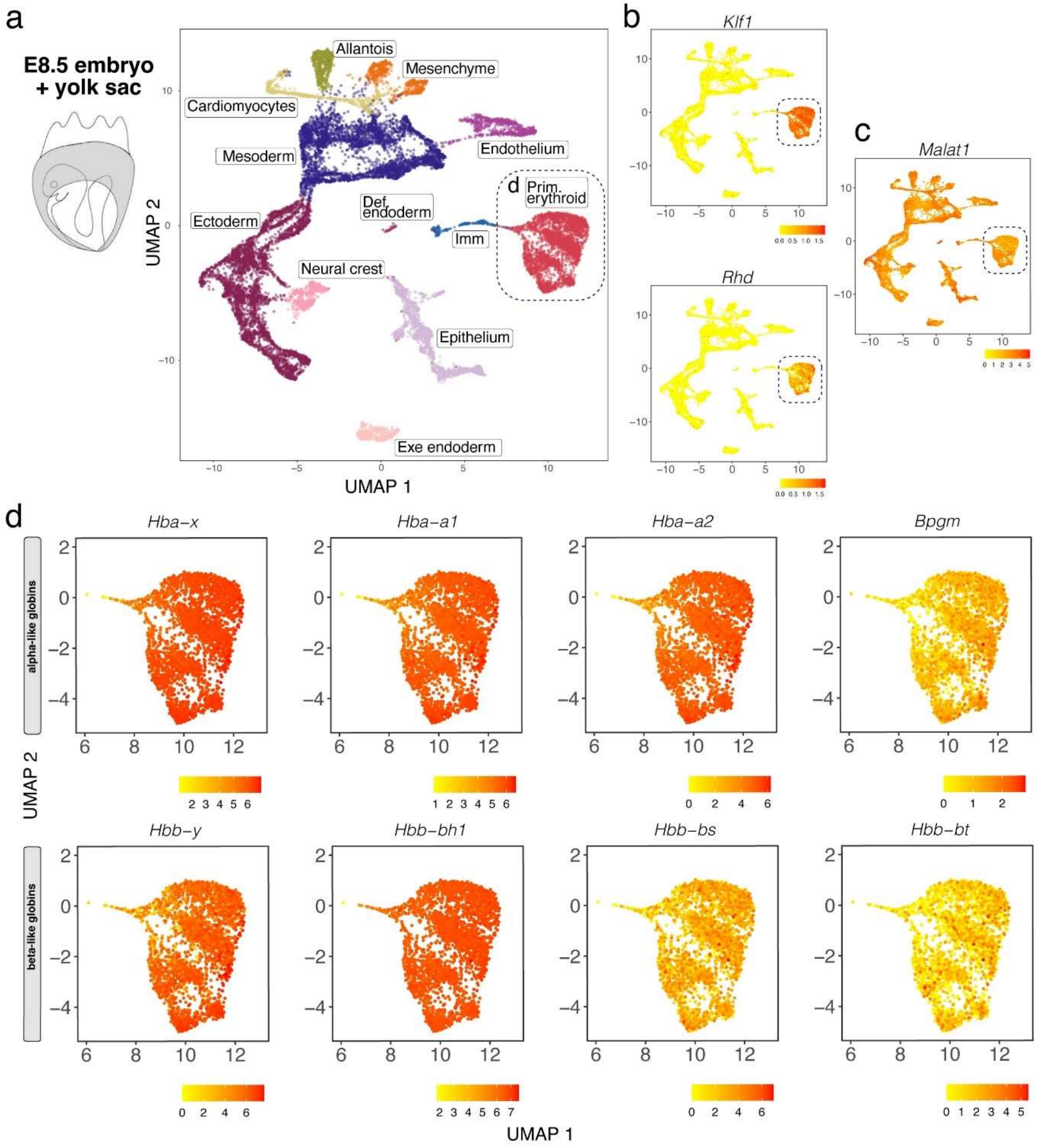

Figure S4. scRNA-seq of E8.5 mouse embryos and their yolk sacs. (**a-c**) UMAP plots visualise clusters of distinct cell types (**a**), the expression of the indicated erythrocyte markers (**b**) and the nuclear marker *Malat1* (**c**) in the total E8.5 embryo and yolk sac; the stippled boxes indicate the erythroid cell subcluster shown in (**d**). (**d**) UMAP plots visualise the expression of the indicated haemoglobin genes and *Bpgm* in the erythroid cell subcluster.

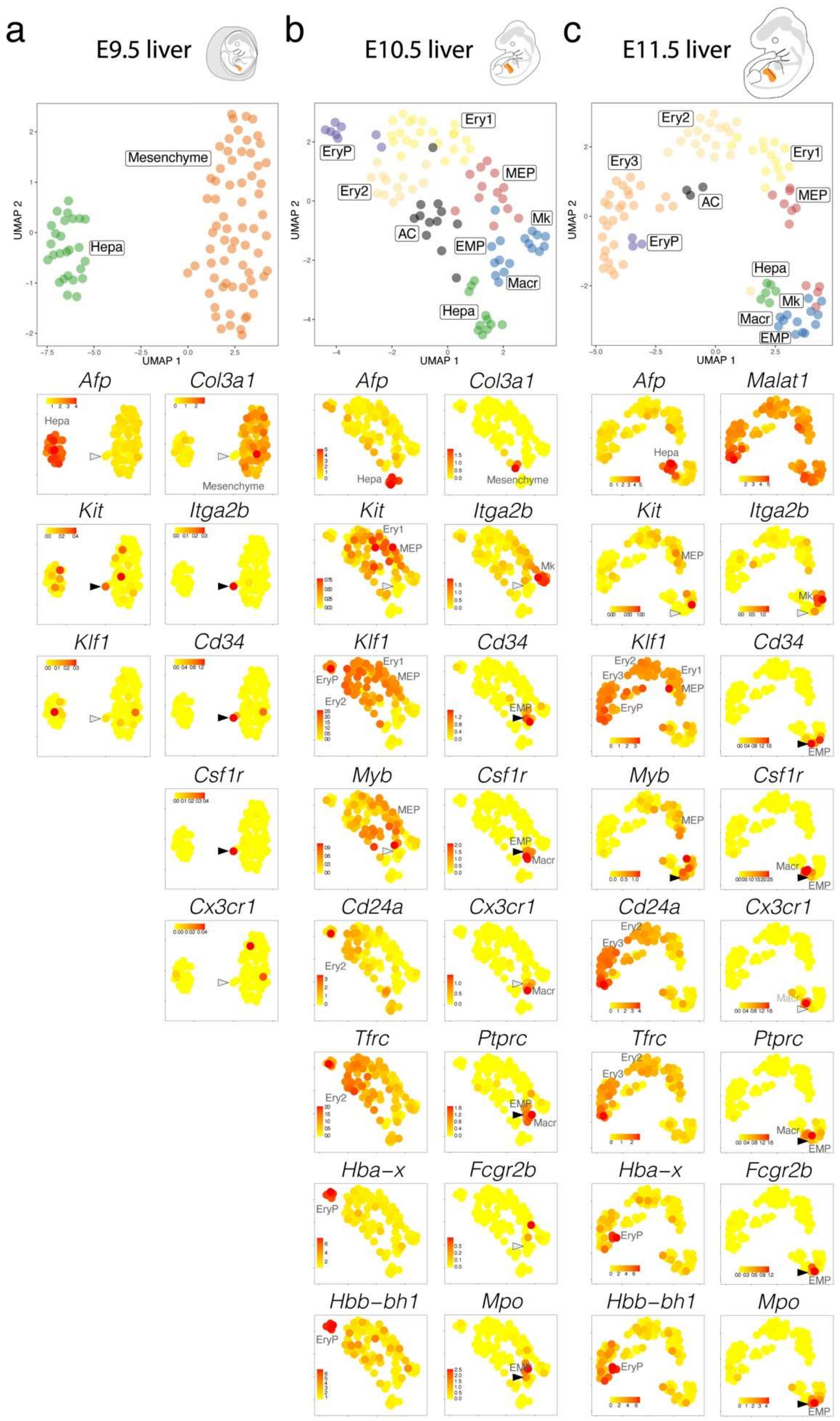

Figure S5. scRNA-seq analysis of E9.5, E10.5 and E11.5 mouse livers. (**a-c**) scRNA-seq time-course of E9.5 (**a**), E10.5 (**b**) and E11.5 (**c**) mouse liver. UMAP plots visualise clusters of distinct cell types (**top panels**), and the expression of the indicated genes (**bottom panels**). AC, apoptotic cells; ND, not detected. Arrowheads and empty arrowheads point to EMPs/MPPs expressing or not the indicated gene, respectively. Each UMAP plot names the cluster(s) expressing the indicated gene.

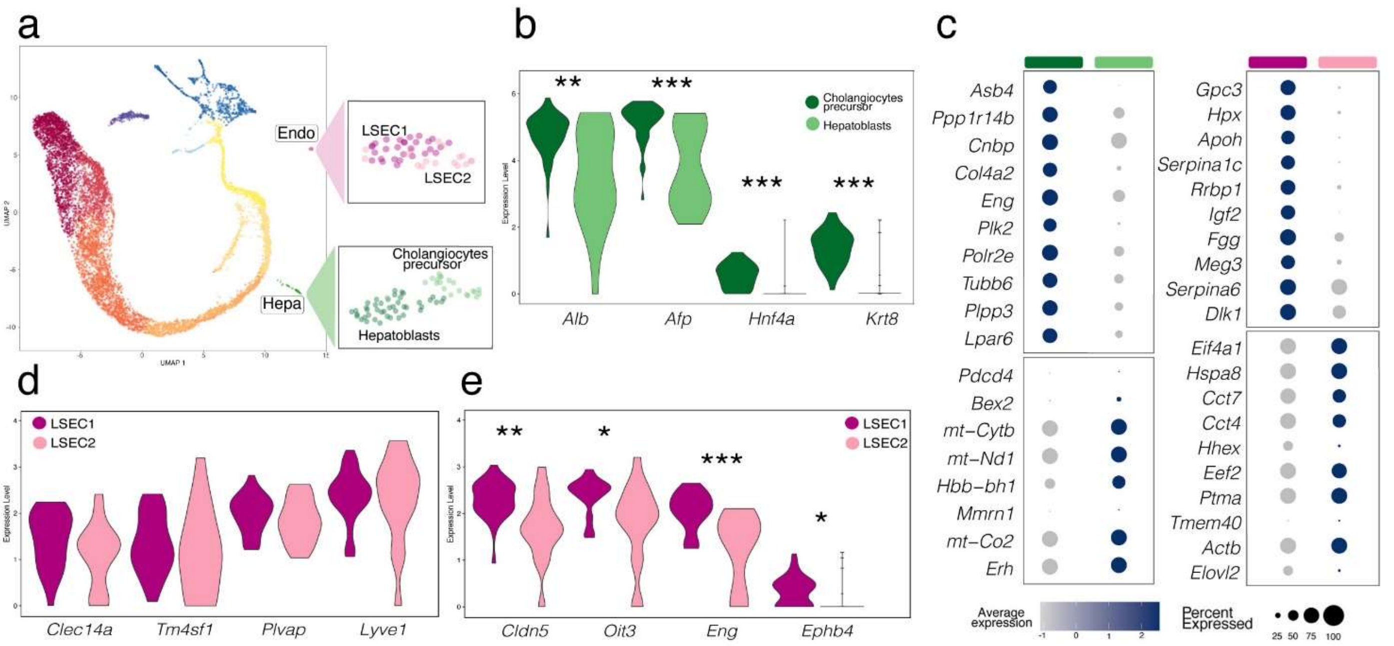

Figure S6. Non-haematopoietic cell identification in E12.5 mouse liver by scRNA-seq analysis. (**a**) Subset selection followed by UMAP plot visualisation identifies subclusters of distinct non-hematopoietic cell types. (**b-e**) Expression of the indicated genes in each subcluster, shown as violin plots (**b,d,e**) and bubble plots (**c**); * p>0.05, ** p<0.01, *** p<0.001 (non-parametric Wilcoxon rank sum test).
